# Quantifying Knowledge Production Efficiency with Thermodynamics: A Data-Driven Study of Scientific Concepts

**DOI:** 10.3390/e28010011

**Published:** 2025-12-22

**Authors:** Artem Chumachenko, Brett Buttliere

**Affiliations:** Centre for European Regional and Local Studies (EUROREG), Science Studies Laboratory, University of Warsaw, Krakowskie Przedmieście 30, 00-927 Warsaw, Poland

**Keywords:** nonequilibrium thermodynamics, thermodynamics of information, Hatano–Sasa decomposition, irreversible work, information efficiency, residual entropy, maximum entropy principle, instantaneous fixed point (IFP), entopy-energy bond, phase transition, finite systems

## Abstract

We develop a data-driven framework for analyzing how scientific concepts evolve through their empirical in-text frequency distributions in large text corpora. For each concept, the observed distribution is paired with a maximum entropy equilibrium reference, which takes a generalized Boltzmann form determined by two measurable statistical moments. Using data from more than 500,000 physics papers (about 13,000 concepts, 2000–2018), we reconstruct the temporal trajectories of the associated MaxEnt parameters and entropy measures, and we identify two characteristic regimes of concept dynamics, stable and driven, separated by a transition point near criticality. Departures from equilibrium are quantified using a residual-information measure that captures how much structure a concept exhibits beyond its equilibrium baseline. To analyze temporal change, we adapt the Hatano–Sasa and Esposito–Van den Broeck decomposition to discrete time and separate maintenance-like contributions from externally driven reorganization. The proposed efficiency indicators describe how concepts sustain or reorganize their informational structure under a finite representational capacity. Together, these elements provide a unified and empirically grounded description of concept evolution in scientific communication, based on equilibrium references, nonequilibrium structure, and informational work.

## 1. Introduction

Entropy occupies a central position in both physics and information theory as a measure of multiplicity, uncertainty, and disorder. Since the work of Boltzmann and Gibbs, thermodynamic entropy has quantified the number of microscopic configurations compatible with a macroscopic state. Shannon [1] extended this concept to symbolic communication, defining information entropy as a measure of uncertainty within message ensembles. Jaynes [2] later showed that the two are not merely analogous but formally connected: statistical mechanics can be reformulated as an inference procedure based on the maximum entropy (MaxEnt) principle, in which physical laws provide the relevant macroscopic constraints. This insight laid the foundation of modern statistical inference and has inspired extensive research linking informational and thermodynamic quantities across physics, chemistry, biology, and cognitive systems [3,4,5].

However, the relationship between thermodynamic and information-theoretic entropy remains a topic of conceptual debate [6,7]. Thermodynamic entropy, rooted in classical physics, measures the logarithm of the number of microstates compatible with a set of macroscopic observables, whereas Shannon entropy quantifies uncertainty associated with a probability distribution. Although their mathematical forms coincide, their physical interpretation aligns only when the probabilities in Shannon’s expression correspond to a MaxEnt equilibrium distribution—the least biased distribution consistent with known constraints. In this sense, Jaynes’s identification of the two entropies is conditional, not universal. As several authors emphasize [8,9], entropy depends on the descriptive level adopted by the observer. Caticha’s entropic-inference framework [10,11] clarifies that the quantity appearing in the exponent of a Boltzmann distribution is simply the observable whose expectation value is constrained in the MaxEnt problem; its interpretation—physical or informational—depends on the modeling context. Gao et al. [12] formalized this connection by showing that thermodynamic and Shannon entropy coincide exactly when the underlying distribution is a generalized Boltzmann distribution, while recent work demonstrates that Clausius entropy can be derived directly from Boltzmann–Gibbs–Shannon entropy under equilibrium assumptions [13]. These results delineate the conditions under which thermodynamic interpretations of informational models are mathematically grounded.

An essential insight from modern nonequilibrium thermodynamics is provided via Landauer’s principle [14], which establishes that logically irreversible transformations of information have an intrinsic thermodynamic cost. In its classical formulation, reducing the Shannon entropy of a system—such as erasing one bit—requires a corresponding increase in environmental entropy, thereby ensuring non-negative total entropy production. Contemporary developments in stochastic thermodynamics [4,5,15,16,17,18,19] interpret this relation as a direct consequence of the second law applied to information-bearing degrees of freedom: any operation that decreases uncertainty must be offset with compensating dissipation. Along with Brillouin’s notion of negentropy [20], Landauer’s principle illustrates a general structure in which entropy balances link informational and thermodynamic descriptions. Although our framework does not involve physical heat flows, these results establish a coherent informational–thermodynamic perspective in which entropy balances constrain transformations of structured information. This perspective motivates the use of entropy-based measures and potential-like quantities when analyzing how empirical information is organized and reorganized in large corpora.

Within this informational–thermodynamic perspective, scientific communication can be regarded as an open informational system in which symbolic units and concepts appear with varying frequency across documents. Because each document provides only a finite representational context, the corpus distributes its limited expressive capacity unevenly among concepts: some receive substantial attention, while others appear only sparsely. This scarcity drives structural constraints that shape how knowledge propagates, leading to the empirical observation that concept-frequency distributions in scientific corpora are consistently heavy-tailed and follow power-laws [21,22,23]. This phenomenon is not unique to text; similar heavy-tailed patterns are endemic to a variety of complex systems, ranging from social collaboration and urban growth to the distribution of wealth and the topology of biological networks [24,25,26].

The maximum entropy (MaxEnt) principle provides the direct theoretical basis for a thermodynamic interpretation of these heavy-tailed distributions. The Random Group Formation (RGF) framework proposed by Baek, Bernhardsson, and Minnhagen [21] is a key model in this context. While RGF uses a complex cost function derived from a mutual entropy minimization procedure, its resulting power–exponential distribution, $N\left(k\right)\propto e^{-bk}/k^{\gamma }$, where N(k) is the number of groups with *k* elements, is analytically derived from a minimal information cost constraint. Crucially, the functional form of this outcome is shown to be equivalent to applying the MaxEnt method with a constraint on the mean logarithmic group size, 〈lnk〉. This logarithmic term, lnk, associated with the group size *k*, quantifies the localization cost required to specify an element within it. This rigorous equivalence is formalized by Visser’s analysis [27], which demonstrates that generalized RGF distributions can be precisely derived from the MaxEnt method for Shannon entropy by applying constraints to both the mean group size, 〈k〉, and the logarithmic group size, 〈lnk〉. Together, these results show that heavy-tailed frequency distributions have a well-defined MaxEnt equilibrium form determined by the first moment and the mean logarithmic size, providing a principled baseline for interpreting empirical distributions.

A few studies have proposed thermodynamic or entropy-based frameworks for analyzing social communication or symbolic systems. Among these, the work by Peng [28] offers an important early attempt to draw an analogy between social collaboration and Boltzmann statistics. However, Peng’s formulation relies on simplifying assumptions that differ from the MaxEnt-based approach adopted here. In particular, the entropy is defined with respect to a combinatorial upper bound, rather than being derived from empirical constraints on the underlying frequency distribution. The logarithmic cost term lnk introduced in the model (where *k* denotes the number of edits to a given Wikipedia page) is chosen to reproduce the observed heavy-tailed pattern, but it does not emerge from a constrained entropy-maximization procedure such as those used in later RGF and MaxEnt formulations. As a consequence, Peng’s model provides a useful qualitative analogy but offers limited guidance for analyzing empirical distributions over time or for characterizing their nonequilibrium evolution under external influences.

A recent approach by Giardini and daCunha [29] addresses time-based evolution by developing a Thermodynamics of Innovation model. However, this framework focuses on cumulative adoption quantities over time, constructing a canonical ensemble to model the temporal evolution of the aggregate population using Gompertz-like and Maxwell–Boltzmann-like shapes. While successful at describing time-series dynamics, this cumulative focus is orthogonal to the need for a frequency-resolved (cross-sectional) analysis of heterogeneous concepts within the literary corpus.

More broadly, the existing approaches do not provide a data-driven, frequency-resolved thermodynamic framework for symbolic communication systems. In particular, no prior work derives equilibrium reference states directly from empirical term-frequency distributions or establishes a principled way to compare these empirical states with their MaxEnt equilibrium counterparts, nor does it model their temporal evolution within an open-system, grand-canonical formalism. Consequently, current models cannot quantify nonequilibrium structure, entropy production, or the thermodynamic efficiency of informational change at the level of individual concepts.

The present work addresses this gap by developing a thermodynamic framework in which each scientific concept is treated as an open, frequency-resolved informational system. The empirical term-frequency distribution defines the concept’s nonequilibrium mesoscopic state, while a MaxEnt-derived generalized Boltzmann distribution provides the corresponding equilibrium reference. The discrepancy between these two distributions—captured via residual entropy and a free-energy–like gap—quantifies the informational structure and stability of each concept. By applying a discrete-time version of the Hatano–Sasa [30] and Esposito–Van den Broeck [4] decomposition, we further separate maintenance-like (housekeeping) contributions from externally driven reorganization. This equilibrium/nonequilibrium pairing is, to our knowledge, new to the study of scientific communication and forms the backbone of our analysis of concept evolution, stabilization, and informational efficiency.

The remainder of the paper is organized as follows. Section 2 introduces the data and formalizes the thermodynamic framework, including the MaxEnt construction of equilibrium reference states, the definition of residual entropy and free-energy measures, and the Hatano–Sasa/Esposito–Van den Broeck decomposition for nonequilibrium evolution. Section 3 presents an empirical analysis of more than 11,000 scientific concepts, detailing their trajectories in thermodynamic state space, identifying characteristic equilibration and driving regimes, and evaluating dissipation and efficiency patterns. Section 4 discusses the theoretical and empirical implications of the proposed framework for understanding concept stabilization and semantic innovation.

## 2. Materials and Methods

### 2.1. Database Description

We used a corpus of 451,524 English-language research articles from the High Energy Physics and Astronomy sections of arXiv (2000–2018). Each document contains full text and standard metadata and was annotated with term-frequency counts for 13,945 scientific concepts drawn from a curated ontology [31,32,33].

Related studies have analyzed this corpus for concept co-occurrence structure, innovation emergence, and topic organization [22,34,35]. Here, we use the same concept-usage data but develop a thermodynamic, MaxEnt-based description, focusing on equilibrium reference states, residual information, and nonequilibrium informational work.

### 2.2. Thermodynamic Framework

To formalize our model, we consider the scientific concept *c* and define its empirical support at time *t* as the collection of relevant documents that contain at least one mention of the concept. The number of such documents is denoted as $N_{c}\left(t\right)$ among the total number of $N_{D}>N_{c}$ documents in the corpus. If no further information is known beyond whether a concept appears in the document or not [28], then a uniform probability, $1/N_{c}\left(t\right)$, can be assigned to each document. This yields a maximum entropy value of $\ln N_{c}\left(t\right)$, representing the highest possible uncertainty under this minimal description [36,37]. In this representation, each document is associated with a single indistinguishable *microstate* of a concept.

More detailed information on a concept state arises from the in-text term frequency analysis, where each frequency class, $k\in Z^{+}$, defines a *mesostate*, a coarse-grained representation of the underlying microstates of individual concept mentions [38]. The corresponding empirical probability, $p\left(k,t\right)=N_{c}\left(k,t\right)/N_{c}\left(t\right)$, is proportional to the number, $N_{c}\left(k,t\right)$, of documents that mention the concept *c* exactly *k* times until time *t*. The Shannon entropy of this mesoscopic description,

(1) $$S\left(t\right)=-\sum_{k=1}^{\infty }p\left(k,t\right)\ln p\left(k,t\right),$$

reflects additional knowledge about the structural organization of p(k,t) and, therefore, is typically smaller than the maximal value $\ln N_{c}\left(t\right)$ corresponding to uniform ignorance.

In the early stage of its appearance, a concept is supported by only a small number of documents, and its mesostate, p(k,t), is correspondingly sparse and almost identical to its microstate configuration. As the concept accumulates sufficient usage across documents, the empirical distribution p(k,t) stabilizes and develops a broad, approximately heavy-tailed form. This enables a power–exponential fit, $p\left(k,t\right)\;\propto \;k^{-\beta_{t}}e^{-\lambda_{t}k}$, defining the concept’s instantaneous fixed point (IFP) [38]. Extensive empirical comparisons with other heavy-tailed distributions performed in our previous work [23] show that this distribution provides the tightest upper envelope (maxentropic bound) across the entire observed range of mesostate Shannon entropies.

As was shown by Visser [27], the distribution parameters β and λ can be obtained from the maximization of Shannon entropy *S* under two empirical constraints: the mean frequency ${\langle k\rangle }_{p}$ and the logarithmic moment ${\langle \ln k\rangle }_{p}$, both calculated from the observed empirical distribution p(k,t). This constrained maximization yields the following [22]:

(2) $$\pi \left(k,t;\beta ,\lambda \right)=\frac{1}{Z}\frac{e^{-\lambda k}}{k^{\beta }},\;Z=\sum_{k=1}^{\infty }\frac{e^{-\lambda k}}{k^{\beta }}=Li_{\beta }\left(e^{-\lambda }\right),$$

where *Z* is the normalization constant expressed through the polylogarithm function $Li_{\beta }\left(e^{-\lambda }\right)$, and β,λ>0 are Lagrange multipliers determined as follows:

(3) $${\langle k\rangle }_{p}={\langle k\rangle }_{\pi }=\frac{Li_{\beta -1}\left(e^{-\lambda }\right)}{Li_{\beta }\left(e^{-\lambda }\right)},\;{\langle \ln k\rangle }_{p}={\langle \ln k\rangle }_{\pi }=-\frac{\partial_{\beta }Li_{\beta }\left(e^{-\lambda }\right)}{Li_{\beta }\left(e^{-\lambda }\right)}.$$

The entropy of the resulting macrostate then follows as [22]

(4) $$\begin{array}{ccc} S_{\mathrm{IFP}} & = & -\sum_{k=1}^{\infty }\pi \left(k,t\right)\ln\pi \left(k,t\right)\\ & = & \ln Z+\;{\langle \ln k\rangle }_{p}+\lambda {\langle k\rangle }_{p}. \end{array}$$

The distribution π(k,t) belongs to the class of generalized Boltzmann distributions, that is, exponential-family distributions obtained as MaxEnt solutions under constraints on 〈lnk〉 and 〈k〉. In the entropic–inference viewpoint [2,10,11], the observables that appear in the exponent play the role of effective “energies”, and the associated Lagrange multipliers are their conjugate intensive parameters. In our case, the constrained quantity E(k)=lnk acts as an internal informational energy, while *k* controls the occupancy of the concept. The mean values

$$U={\langle \ln k\rangle }_{\pi },\;N={\langle k\rangle }_{\pi }$$

therefore represent, respectively, the internal informational energy and the mean number of logical particles (concept mentions) in the ensemble. The normalization constant *Z* plays the role of a generalized partition function for this open informational system.

For systems described by generalized Boltzmann distributions, the Gibbs–Shannon entropy coincides (up to an additive constant) with the thermodynamic entropy in the formal sense demonstrated in Refs. [12,13,39]. Under this formal equivalence, it is convenient to introduce effective intensive parameters

$$T=\frac{1}{\beta },\;\mu =-\;\frac{\lambda }{\beta },$$

which mirror, at the level of Legendre structure, the roles played by temperature and chemical potential in physical thermodynamics. We emphasize that *T* and μ here are *informational* intensive parameters, not physical quantities.

With this notation, the thermodynamic–like potential takes the standard grand-canonical form

(5) $$S_{\mathrm{therm}}=k_{B}S_{\mathrm{IFP}}=k_{B}\left(\ln Z+\beta U-\mu \beta N\right),$$

providing a consistent set of state variables for analyzing the equilibrium and nonequilibrium properties of concept–frequency distributions.

The notion of internal equilibrium is essential: although concept usage evolves over time and the empirical distribution is rarely at equilibrium globally, within each time window, the IFP defines a locally equilibrated reference state that maximizes entropy under the empirical constraints. This is precisely the regime in which generalized Boltzmann distributions acquire a consistent thermodynamic structure [12,39,40].

### 2.3. Residual Entropy and Free Energy

We refer to a concept as being in *equilibrium* when its empirical entropy, *S*, coincides with the corresponding MaxEnt value $S_{\mathrm{IFP}}$. In this regime, the macroscopic descriptors (U,N,T,μ) inferred from the MaxEnt distribution remain effectively constant, and the informational structure of the concept is stabilized.

Departures from this equilibrium reference are quantified by the residual entropy

(6) $$R\left(t\right)=S_{\mathrm{IFP}}\left(t\right)-S\left(t\right)=D_{\mathrm{KL}}\;\left(p(t)∥\pi (t)\right)=\sum_{k}p\left(k,t\right)\ln\;\frac{p(k,t)}{\pi (k,t)}\geq 0,$$

which measures the additional information required to specify the empirical state relative to its MaxEnt equilibrium projection [22]. In this sense, *R* plays the role of an information distance from the IFP manifold.

To analyze this nonequilibrium structure, we introduce the corresponding *informational* grand potentials,

(7) $$\Phi_{\mathrm{IFP}}=-T\ln Z,\;\Phi =U_{p}-TS-\mu N_{p},$$

which follow the usual Legendre form of generalized Boltzmann ensembles. These quantities are not physical energies or work; rather, they are formal thermodynamic potentials arising from the MaxEnt representation of the concept distribution.

Using Equations (3), (6) and (7), the difference between equilibrium and non-equilibrium informational grand potentials at fixed (T,μ) or, equivalently, (U,N) satisfies the following:

(8) $$\Phi -\Phi_{\mathrm{IFP}}=T\;R\geq 0.$$

This is the exact information-theoretic analogue of the nonequilibrium free-energy identity in stochastic thermodynamics [4]: the excess informational grand potential equals the residual entropy multiplied by the intensive parameter *T*. The identity in Equation (8) has the same mathematical structure as the Landauer-type relation: reductions in uncertainty (here represented as *R*) require a corresponding decrease in a potential-like quantity. In physical systems, this manifests as heat dissipation; in our formal, informational setting it quantifies the minimal “informational work” needed to maintain structure beyond the MaxEnt equilibrium. Thus, the relation provides a Landauer-type interpretation without assuming any physical energy flows.

Across a finite interval, Δt, between initial (*i*) and final (*f*) states, the change

(9) $$\Delta R=\Delta S_{\mathrm{IFP}}-\Delta S=R^{f}-R^{i}$$

captures how the concept’s nonequilibrium organization evolves. This form is directly analogous to the generalized second-law expressions of Esposito and Van den Broeck, which separate changes in entropy into equilibrium and nonequilibrium components in Landauer-like identities. As the mesostate distribution p(k,t) is updated through new documents, ΔR typically decreases, but *S* and $S_{\mathrm{IFP}}$ may vary non-monotonically during semantic innovation or attention shifts. Temporary increases in *R* reflect transient reorganization, rather than a violation of the long-term relaxation trend.

At a fixed *T*, the corresponding change in excess potential satisfies

(10) $$\Delta {\left(\Phi -\Phi_{\mathrm{IFP}}\right)}_{T}\approx T\;\Delta R,$$

so that decreases in *R* correspond to decreases in the excess informational grand potential, consistent with relaxation toward the IFP manifold whenever the empirical state moves closer to its MaxEnt reference.

### 2.4. Dissipation Towards a Stationary Reference State

As a baseline, we first consider the regime in which the instantaneous fixed point π is stationary and the thermodynamic control parameters (T,μ) remain constant. Under these conditions, the empirical distribution relaxes towards its IFP, p→π, in the absence of external driving.

For such isothermal, fixed-μ transitions, the integrated second law can be written in the Esposito–Van den Broeck form [4]:

(11) $$W_{\mathrm{irr}}=T\;\Delta S_{i}+T\;\Delta R,$$

where $\Delta S_{i}\geq 0$ is the irreversible entropy production [4,15]. This identity follows from the standard stochastic thermodynamic decomposition of entropy production and work for open systems [4,15]; a detailed derivation adapted to our notation is provided in Appendix B.

When π is stationary, local detailed balance implies (see Appendix C)

$$\frac{dS_{i}}{dt}=-\;\frac{dR}{dt},$$

which integrates over a relaxation interval to

(12) $$\Delta S_{i}=-\Delta R,\;\Rightarrow \;W_{\mathrm{irr}}=0.$$

This result characterizes the simplest form of conceptual dynamics: In the absence of external driving, any departure of *p* from its fixed point is dissipated entirely as entropy production, and the residual entropy R(t) monotonically decreases. In this regime, R(t) behaves as a Lyapunov function, governing the convergence of the empirical distribution to the IFP manifold [15,18,41,42].

In physical systems, this regime corresponds to standard isothermal relaxation processes: with fixed intensive parameters (T,μ), probability mass is redistributed across microstates until the stationary distribution is reached, while the irreversible work $W_{\mathrm{irr}}$ vanishes over the full relaxation process. In our informational setting, an analogous behavior is observed for concepts whose empirical distributions remain close to a fixed IFP and whose inferred intensive parameters (T,μ) vary only weakly over time. Such concepts operate in a regime of thermodynamic buffering, where incoming documents primarily refine the sampling of an essentially stationary reference distribution, rather than driving the system away from it.

As we show below, this buffering behavior can be understood in terms of the geometry of the MaxEnt manifold. Concepts whose fitted parameters (β,λ) place them near the heavy–tailed region of the parameter space (β≈3/2,λ≈0) exhibit markedly increased thermodynamic response coefficients. In this region, the curvature of the MaxEnt surface becomes large, making the macroscopic quantities (U,N) highly sensitive to small perturbations of (T,μ). Empirical concepts rarely reach the singular power–law limit λ→0, but many lie close enough to it to display a characteristic “critical-like” enhancement of heat capacity and susceptibility. Paradoxically, this heightened responsiveness enables certain concepts to keep their intensive parameters nearly constant: fluctuations in (U,N) are absorbed without substantial movement in (T,μ), producing the observed thermodynamic buffering. In this sense, the equilibrated portion of the vocabulary acts as an effective informational reservoir.

Within this framework, the cutoff λ sets the effective extent of accessible frequency states, while β controls their scaling and thereby governs the proximity to the heavy–tailed regime. To quantify the resulting degrees of responsiveness, we consider the standard thermodynamic response coefficients associated with the MaxEnt model.

The heat capacities

(13) $$C_{\mu }={\left(\frac{\partial U}{\partial T}\right)}_{\mu },\;\alpha_{U}={\left(\frac{\partial U}{\partial \mu }\right)}_{T},$$

describe how the informational energy *U* responds to variations in the intensive parameters, while the susceptibilities

(14) $$\chi_{T}={\left(\frac{\partial N}{\partial \mu }\right)}_{T},\;\alpha_{\mu }=\frac{1}{N}\;{\left(\frac{\partial N}{\partial T}\right)}_{\mu },$$

characterize the corresponding response of the mean concept usage *N*.

Because all of these coefficients are derived from the same grand-potential function, they share the same qualitative structure and exhibit parallel signatures near the heavy–tailed region of the MaxEnt manifold. For empirical analysis, it is, therefore, sufficient to work with a reduced pair of combined differential coefficients that capture the joint variations of both *U* and *N*. To this end, we define the effective energy E=U−μN, whose differential

(15) $$dE={\left(\frac{\partial E}{\partial T}\right)}_{\mu }\;dT+{\left(\frac{\partial E}{\partial \mu }\right)}_{T}\;d\mu =C_{\mu }^{E}\;dT+\alpha_{E}\;d\mu ,$$

provides a compact summary of responsiveness through the coefficients $\left(C_{\mu }^{E},\alpha_{E}\right)$. These quantities behave analogously to the full set of heat capacities and susceptibilities but offer a clearer geometric interpretation on the (T,μ) manifold.

Large values of $\left(C_{\mu }^{E},\alpha_{E}\right)$ identify concepts with enhanced responsiveness—characteristic of systems approaching the heavy–tailed boundary—where extensive quantities exhibit substantial fluctuations while intensive parameters vary only weakly. In contrast, dynamically driven concepts typically appear *before* reaching these critical regions: their IFP trajectories (T(t),μ(t)) begin to deviate from the stationary manifold while the response coefficients are still rising but have not yet diverged. This pre-critical regime reflects the onset of semantic reorganization, where changes in topic context or conceptual usage start to generate irreversible work, $W_{\mathrm{irr}}>0$, before full buffering behavior is lost. The next subsection examines this driven regime in detail and quantifies how much of the resulting dissipation supports structural adaptation versus the maintenance of the existing conceptual organization.

### 2.5. Dynamic Efficiency and Non-Equilibrium Work Decomposition (Driving Case)

To analyze concept evolution under external informational driving, we now make explicit the discrete-time structure that was implicit in the preceding discussion. We represent the corpus as a sequence of snapshots, $t_{0}<t_{1}<\cdots <t_{f}$, with each corresponding to an observational window (e.g., one year). At every snapshot, $t_{i}$, we compute the empirical mesostate $p_{t_{i}}\left(k\right)$ and its instantaneous fixed point (IFP), $\pi_{t_{i}}\left(k\right)$, obtained from the MaxEnt construction described above. Each step, $t_{i}\to t_{i+1}$, therefore constitutes a transition between coarse-grained nonequilibrium steady states (NESSs) in the sense of Hatano–Sasa [30] and the Esposito–Van den Broeck framework for Markovian thermodynamics [4,15]. The resulting trajectory, $\left\{p_{t},\pi_{t}\right\}$, captures how the internal energy, U(t), and particle number, N(t), reorganize in response to external informational inputs.

In the Esposito–Van den Broeck framework, the total irreversible entropy production generated via a finite driven evolution admits the decomposition

(16) $$\Delta S_{\mathrm{tot}}=\Delta S_{\mathrm{na}}+\Delta S_{a}\geq 0.$$

The non-adiabatic component $\Delta S_{\mathrm{na}}$ quantifies entropy generated via changes in the IFP parameters (structural or contextual adaptation), while the adiabatic component $\Delta S_{a}$ corresponds to the steady “house-keeping” dissipation required to maintain the nonequilibrium structure when the parameters are held fixed. In the absence of driving (stationary $\pi_{t}$), this reduces to the intrinsic entropy production discussed earlier.

For discrete-time dynamics, the non-adiabatic contribution can be written in terms of changes in residual entropy change, together with an excess term associated with parameter driving. Following the Esposito–Van den Broeck Markov formulation (see Appendix D for derivation), we obtain

(17) $$\Delta S_{\mathrm{na}}=-\Delta R+\sum_{t}{\hat{Y}}_{t\to t+1},\;\Delta S_{a}=\Delta S_{\mathrm{tot}}-\Delta S_{\mathrm{na}},$$

where −ΔR≥0 is the boundary term produced via changes in the KL divergence $D_{\mathrm{KL}}\left(p_{t}∥\pi_{t}\right)$, and

(18) $${\hat{Y}}_{t\to t+1}=\sum_{k}p_{t}\left(k\right)\;\ln\frac{\pi_{t}\left(k\right)}{\pi_{t+1}\left(k\right)}$$

is the ensemble-averaged excess contribution generated purely via changes in the IFP parameters (T(t),μ(t)). This term is the discrete counterpart of the Hatano–Sasa excess functional for driven steady states.

Using (16) and (17) yields the driving/housekeeping split [30]:

(19) $$W_{\mathrm{driving}}=T_{\mathrm{ref}}\;\Delta S_{\mathrm{na}},\;W_{\mathrm{hk}}=T_{\mathrm{ref}}\;\Delta S_{a},\;W_{\mathrm{irr}}=W_{\mathrm{ex}}+W_{\mathrm{hk}}.$$

Thus, $W_{\mathrm{ex}}$ quantifies the energetic cost of structural adaptation to changing semantic conditions, while $W_{\mathrm{hk}}$ represents steady-state dissipation required to maintain the nonequilibrium distribution.

The reference temperature $T_{\mathrm{ref}}$ specifies the statistical environment (bath) for evaluating work quantities. We use either (i) a global reference $T_{⋆}$ obtained from the ontology-wide mode, or (ii) a topic-specific temperature $T_{\mathrm{core}}$ estimated from equilibrated core concepts. Because efficiencies depend only on entropy-production ratios, all reported efficiency measures are dimensionless and independent of the choice of $T_{\mathrm{ref}}$.

To evaluate how dissipation partitions between maintenance and adaptation, we introduce the dimensionless efficiencies

(20) $$\eta_{\mathrm{hk}}=\frac{\Delta S_{a}}{\Delta S_{\mathrm{tot}}},\;\eta_{\mathrm{ex}}=\frac{\Delta S_{\mathrm{na}}}{\Delta S_{\mathrm{tot}}}=1-\eta_{\mathrm{hk}}.$$

In addition, we define the residual–information ratio

(21) $$\rho_{R}=\frac{{\left[-\Delta R\right]}_{+}}{\Delta S_{\mathrm{tot}}},$$

which quantifies the fraction of total entropy production that is effectively used to reduce the residual informational structure. This measure is analogous in spirit to the information–erasure efficiencies introduced by Allahverdyan et al. [43].

High values of $\eta_{\mathrm{hk}}$ indicate stabilized concepts whose evolution is dominated by steady maintenance, while high $\eta_{\mathrm{ex}}$ identifies adaptive concepts whose informational structure is being reorganized over the interval Δt.

These two regimes manifest equally clearly in the behavior of the residual–information ratio $\rho_{R}$. In the absence of external driving, relaxation proceeds entirely through the elimination of residual entropy, so $\rho_{R}$ attains its maximal value (Equation (12)). Under driving, however, part of the dissipation is diverted toward accommodating changes in the evolving IFP, leading to reduced values of $\rho_{R}$. Thus, $\rho_{R}\approx 1$ signals simple relaxation toward an equilibrium, whereas smaller values reveal periods of genuine adaptive reorganization in response to shifts in the semantic environment.

## 3. Results

For each of the 13,945 concepts in the ontology, we estimated the MaxEnt parameters (β,λ) of the macrostate (IFP) distribution (Equation (2)) using maximum-likelihood estimation. To ensure internal consistency, each fitted pair was required to reproduce the empirical moment constraints 〈k〉 and 〈lnk〉 (Equation (3)) within a numerical tolerance of $10^{-5}$. Concepts with a small number of mentions provided insufficient information for stable parameter inference and were excluded; over 2000–2018, this affected 2208 early-stage concepts, leaving 11,737 analyzable cases.

**Figure 1:**
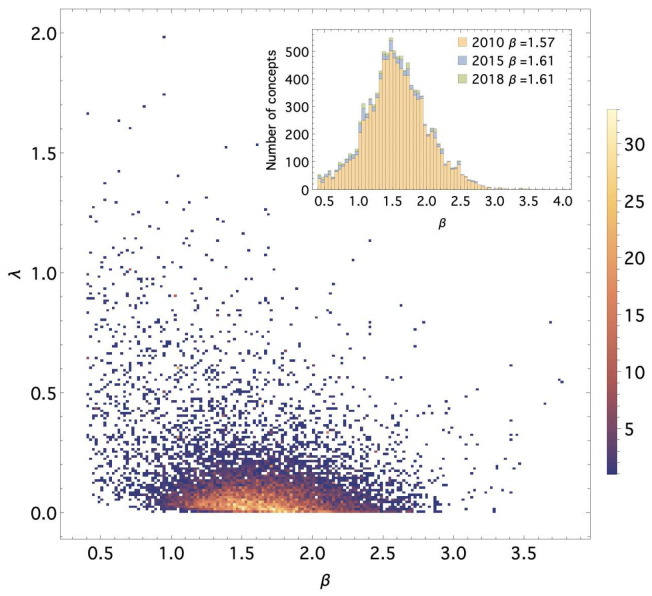
Empirical distribution of fitted MaxEnt parameters (β,λ) for 11,737 scientific concepts across 2000–2018. Panels show the corpus-wide distribution of β over selected time periods and highlight the persistent concentration of values near β≃1.5.

Figure 1 shows the empirical distribution of fitted (β,λ) values across all concepts for the period of 2000–2018. Most estimates lie within the region β∈[1,2] and λ∈[0,0.15], with a persistent mode near β≃1.5. Time-averaged values of β remain remarkably stable ($\bar{\beta }\approx 1.51$ through 2010 and $\bar{\beta }\approx 1.61$ in 2018), consistent with the heavy-tailed usage patterns typical of scientific terminology [22].

To characterize broad equilibration patterns, we examined how the fitted parameters relate to the following: (i) the time at which a concept first appears and (ii) the number of documents supporting it. Three robust empirical regularities emerge.

First, concepts introduced earlier in the corpus more frequently exhibit small residual entropy *R*, indicating close agreement between empirical usage distributions and the MaxEnt prediction. Recently introduced concepts also reach this regime once sufficient document support accumulates.

Second, the number of supporting documents $N_{c}$ is a strong predictor of equilibration. Across all years, most concepts with $N_{c}≳10^{3}$ satisfy R≈0, and the empirical entropy *S* nearly coincides with the macrostate entropy $S_{\mathrm{IFP}}$. As support increases, the fitted parameter λ typically decreases, yielding heavier-tailed usage consistent with the λ→0 limit.

Third, equilibrated high-support concepts tend to exhibit higher mesoscopic entropy *S* and internal energy *U*, while their grand potential Φ is lower. This reflects a reduction in the residual information potential TR as concepts stabilize. Newly introduced or sparsely used concepts remain underdetermined in short windows but become analyzable as more evidence accumulates; for example, the *Anomalous Hall effect* required approximately four years of accumulation (2000–2004) before the empirical moments could be reliably matched.

Taken together, these findings reveal a coherent empirical structure underlying concept evolution. Most concepts converge toward a narrow region of parameter space centered near β≃1.5; the exponential cutoff parameter λ decreases systematically with increasing support; and the residual entropy *R* falls sharply once a concept exceeds roughly $10^{3}$ supporting documents. A subset of long-established, high-support concepts forms a stable background whose distributions already satisfy the MaxEnt constraints, providing a statistically coherent reference against which more adaptive concepts evolve.

### 3.1. Energy–Entropy Diagram

**Figure 2:**
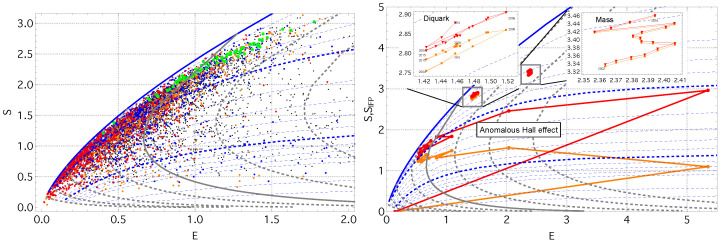
(**left**) Energy–entropy diagram for 11,737 concepts (2018). The green points denote stationary (equilibrated) concepts. The blue (R<0.04) and orange (R<0.004) points identify concepts with low residual entropy, while the red points highlight concepts with a near power-law term-frequency spectrum (λ<0.04). The black points represent all remaining concepts. The dashed blue and gray curves are theoretical MaxEnt envelopes, $S_{\mathrm{IFP}}\left(E;\lambda ,\beta \right)$, for fixed cutoff values, λ>0 and fixed β>0, respectively. The dashed gray curves show isotherms for β∈{0.5,1,2}, with the solid gray curve marking β=1.5. The solid blue curve represents the heavy-tailed limit λ→0, here evaluated at $\lambda =10^{-3}$. (**right**) The trajectories of three representative concepts—*Diquark*, *Mass*, and the *Anomalous Hall Effect*. The orange lines show the empirical mesostate evolution, while the red lines show the corresponding IFP trajectory.

To examine the global organization of concept states, we embed each concept in the energy–entropy (E–S) plane using the effective energy E=〈lnk−μk〉 and the empirical mesostate entropy *S*. Figure 2 (left) shows the resulting diagram. All concepts lie below the theoretical maximum for the macrostate entropy $S_{\mathrm{IFP}}\left(E\right)$, represented by the solid blue line and computed from Equation (4) in the near–power-law limit $\lambda =10^{-3}$ as a function of effective energy *E*. The dashed blue curves show the corresponding theoretical envelopes $S_{\mathrm{IFP}}\left(E\right)$ for larger cutoff values $\lambda >10^{-3}$ (in increments of 0.01), illustrating how finite-size effects progressively suppress the maximum attainable entropy at a fixed *E*.

Figure 2 (right) illustrates representative trajectories of concept state evolution. *Diquark* and *Mass* both follow nearly stationary paths for more than a decade, maintaining almost constant effective energy *E* and macrostate entropy $S_{\mathrm{IFP}}$. The *Diquark* trajectory shows a gradual decrease in both empirical mesostate entropy *S* and residual entropy *R*, accompanied by small oscillations in the inferred temperature. In contrast, *Mass*, probably the most basic concepts in this dataset, remains extremely close to its IFP at all times (ΔR≃0), exhibiting only a modest increase in *S* and $S_{\mathrm{IFP}}$ while keeping *E* essentially unchanged.

**Figure 3:**
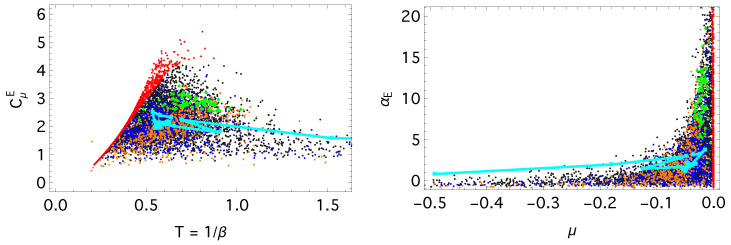
Heat capacity, $C_{\mu }^{E}$, and susceptibility, $\alpha_{E}$, diagrams from Equation (15). The cyan curve traces the trajectory of the *Anomalous Hall Effect* concept; the trajectory terminates at the year 2018, when its estimated temperature reaches T≃0.6 and μ≃−0.05. The color of the points denotes concept classes, as in Figure 2.

The *Anomalous Hall effect* concept illustrates a typical emergence trajectory. Initially, both *T* and μ fluctuate strongly, indicating a driving regime in which the concept adapts to a rapidly changing topical environment. As the concept stabilizes, these fluctuations diminish, and the trajectory approaches the β≃1.5 isotherm, entering a non-driving regime in which the evolution becomes effectively autonomous. This region coincides with maximal heat capacity and susceptibility (Figure 3), reflecting a buffered state where intensive variables remain stable despite continued information exchange.

Figure 3 displays the effective response functions $C_{\mu }^{E}$ and $\alpha_{E}$, which are representative of the broader family of thermal and chemical susceptibilities introduced in Equations (13) and (14). To relate these responses to the global entropic structure of the system, we introduce a dimensionless intensive entropy–energy ratio that empirically remains almost constant for equilibrated concepts. This ratio was previously discussed as an *entropy reduction ratio* by Peng et al. [28]:

(22) $$TQ^{\mathrm{eff}}=\frac{TS_{\mathrm{IFP}}}{E}=\frac{E-\Phi_{\mathrm{IFP}}}{E},$$

which quantifies how much equilibrium entropy a concept produces per-unit effective energy, *E*, times the local temperature, *T*. The second equality follows directly from Equations (5) and (7), using the thermodynamic identity $\Phi_{\mathrm{IFP}}=E-TS_{\mathrm{IFP}}$.

**Figure 4:**
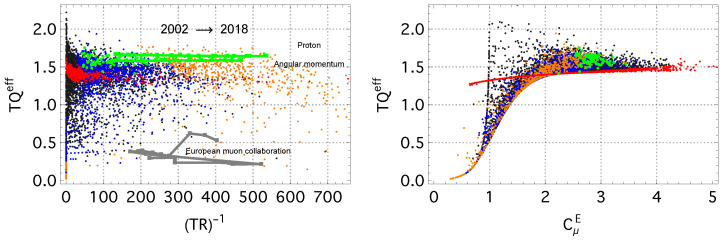
(**left**) The values of the $TQ^{\mathrm{eff}}$ as a function of the (inverse) residual free energy ${\left(TR\right)}^{-1}$ (Equation (8)). Trajectories for the equilibrium concepts of the *Proton* and the *Angular momentum* are shown in green; the trajectory for the driven concept *European muon collaboration* is shown in a gray color. (**right**) The intensive entropy–energy ratio $TQ^{\mathrm{eff}}$ as a function of the heat capacity $C_{\mu }^{E}$ (Equation (15)). The scatter plot for the period of 2002–2018 with the color scheme as that in Figure 2.

Empirically, all equilibrated concepts cluster around a characteristic value, $TQ^{\mathrm{eff}}\;≃\;1.5$ (green points in Figure 4). Their trajectories in the $\left({\left(TR\right)}^{-1},TQ^{\mathrm{eff}}\right)$ plane remain close to this level and typically follow nearly horizontal iso–$TQ^{\mathrm{eff}}$ curves. Such behavior reflects monotonic relaxation toward the IFP manifold at a constant temperature, *T*: the residual entropy decreases (ΔR<0), together with the non-equilibrium free-energy difference $\Delta (\Phi -\Phi_{\mathrm{IFP}})$ (Equation (10)), and the irreversible entropy production remains positive. In such a stationary-reference regime, the relation $\Delta S_{i}=-\Delta R$ holds, so these contributions cancel, and the net irreversible work satisfies $W_{\mathrm{irr}}=0$ over the full relaxation interval.

By contrast, driven concepts exhibit deviations of various amplitudes away from the $TQ^{\mathrm{eff}}≃1.5$ band, during which both *R* and $Q^{\mathrm{eff}}$ can temporarily deviate from their relaxation trend. These episodes signal adaptive reorganization of the usage distribution and are accompanied by Hatano–Sasa excess entropy production, indicating genuine non-equilibrium driving.

For concepts whose IFP distributions approach the power–law limit (red points in Figure 4), the quantity $TQ^{\mathrm{eff}}$ approaches a well–defined empirical plateau. Across the corpus, this plateau lies near $TQ_{\mathrm{crit}}^{\mathrm{eff}}\approx 1.5$, and it is associated with concepts whose thermodynamic parameters lie close to the empirical critical region around $\beta ≃\beta_{c}=1.5$ (cf. Figure 3). To highlight this connection, Figure 4 (right) presents the state diagram in $\left(C_{\mu }^{E},\;TQ^{\mathrm{eff}}\right)$ coordinates, where the high-$TQ^{\mathrm{eff}}$ plateau aligns with the region of maximal response coefficients.

Most equilibrated concepts (green points) align with this plateau. This deviation reflects finite–size effects associated with strictly positive cutoff values λ, which limit the accessible frequency range and keep equilibrated concepts away from the heavy–tailed regime where the plateau is attained.

A useful way to interpret the observed plateau in the entropy–energy ratio $TQ^{\mathrm{eff}}$ is by analogy with classical scaling relations in statistical physics, where entropy and energy often exhibit approximately linear dependencies over characteristic regions of the state space. For example, in bounded thermodynamic systems and in hadronic matter near the Hagedorn transition, the entropy grows proportionally to energy up to a characteristic scale determined by the underlying distribution of microstates [44,45]. In our informational setting, the empirical concentration of equilibrated concepts around a nearly constant value of $TQ^{\mathrm{eff}}$ reflects an analogous finite-size scaling behavior: the informational “capacity” of concepts stabilizes, with equilibrated concepts generating equilibrium entropy in a near-constant proportion to their effective energy. Departures from this level marks adaptive reorganization and non-equilibrium driving, during which both *R* and $TQ^{\mathrm{eff}}$ may temporarily increase or decrease.

### 3.2. Dissipative Regimes and Efficiency of Informational Maintenance

The entropy–production decomposition introduced in Section 2.5 allows us to quantify how irreversible dissipation is partitioned between two components: (i) the housekeeping contribution $W_{\mathrm{hk}}$, associated with sustaining the current nonequilibrium structure and (ii) the excess contribution $W_{\mathrm{driving}}$, arising from changes in the instantaneous fixed-point (IFP) parameters (T,μ) during externally driven evolution. The corresponding dimensionless ratios from Equation (20) characterize the relative weight of these two types of dissipation, while the residual–information ratio $\rho_{R}$ (Equation (21)) measures the extent to which entropy production offsets changes in the residual entropy, *R*.

**Figure 5:**
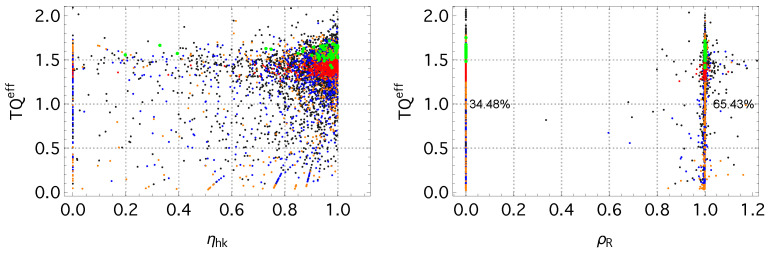
(**left**) The product $TQ^{\mathrm{eff}}$ plotted against the housekeeping efficiency $\eta_{\mathrm{hk}}$ for the 2017–2018 interval. (**right**) The intensive energy–entropy ratio $TQ^{\mathrm{eff}}$ versus the residual–information ratio $\rho_{R}$. Color denotes the concept class, as in previous figures.

Figure 5 (left) displays the empirical distribution of $\eta_{\mathrm{hk}}$ for the 2017–2018 interval. A large fraction of concepts cluster near $\eta_{\mathrm{hk}}\;≃\;1$, indicating that most irreversible dissipation in this period is classified as housekeeping, rather than excess. Equilibrated concepts (green points) concentrate mostly near $\eta_{\mathrm{hk}}=1$. Concepts with nearly power-law usage spectra (λ<0.005) also predominantly lie in this region. The residual entropy value alone does not correlate strongly with $\eta_{\mathrm{hk}}$, reflecting that $\eta_{\mathrm{hk}}$ depends on the relative magnitudes of adiabatic and non-adiabatic entropy production, rather than on the instantaneous distance from equilibrium.

The residual–information ratio $\rho_{R}$ exhibits a characteristic clustering around two values, approximately 0 and 1 (Figure 5 (right)). When $\rho_{R}\approx 0$, the residual entropy remains essentially unchanged (ΔR≈0), so no dissipation is required to compensate for changes in informational structure; all entropy production contributes to $\Delta S_{\mathrm{tot}}$. In contrast, the ridge at $\rho_{R}=1$ corresponds to the case $\Delta S_{\mathrm{tot}}=\left|\Delta R\right|$, characteristic of non-driven relaxation where entropy production precisely offsets the decrease in residual entropy.

In the 2017–2018 window, most concepts lie on the $\rho_{R}\approx 1$ ridge (65.4%), with the remainder at $\rho_{R}\approx 0$. Equilibrated concepts appear on both ridges: some exhibit ΔR≈0 and thus fall at $\rho_{R}\approx 0$, while others undergo slow, undriven relaxation and satisfy $\Delta S_{\mathrm{tot}}=\left|\Delta R\right|$, placing them at $\rho_{R}=1$. Concepts with large accessible frequency ranges (high $k_{\max}$, i.e., small λ) are also largely confined to the two ridges. More adaptively evolving concepts show limited dispersion around $\rho_{R}≃1$, indicating intermittent deviations from non-driven relaxation dynamics.

Overall, the empirical patterns indicate that concept evolution can be grouped into three broad operational regimes based on dissipation characteristics: (i) a *maintenance-dominated regime* ($\eta_{\mathrm{hk}}\;\approx \;1$, $\rho_{R}\approx 0$), where concepts exhibit minimal changes in residual structure; (ii) a *balanced relaxation regime* ($\rho_{R}=1$), in which the entropy production and the residual entropy change are of a comparable magnitude; and (iii) an *adaptive regime* ($\eta_{\mathrm{hk}}<1$, $\rho_{R}\neq 1$), characterized by significant excess dissipation and pronounced variation in the IFP parameters. This classification offers a concise, data-driven picture of how concepts allocate irreversible dissipation across different phases of their evolution.

## 4. Discussion

The frequency distributions of scientific concepts exhibit the heavy-tailed behavior characteristic of many symbolic and social systems. Following Baek et al. [21], Visser [27], and Martini et al. [22], we model these distributions using a maximum entropy (MaxEnt) framework constrained by the empirical moments 〈k〉 and 〈lnk〉. The logarithmic moment reflects the information cost of locating a concept within a frequency class, and under these constraints, the MaxEnt solution takes the geometric–power-law form

$$p\left(k\right)\propto k^{-\beta }e^{-\lambda k},$$

which can be written as a generalized Boltzmann distribution with an effective internal energy of U(k)=lnk and chemical potential μ. This provides a principled equilibrium reference state for each concept, grounded directly in the observed frequency data.

The empirical frequency distribution defines a concept’s nonequilibrium mesoscopic state, while the corresponding MaxEnt solution represents its equilibrium projection. Their divergence, quantified by the residual entropy (Equation (6)), captures informational structure not explained by the MaxEnt constraints. Residual entropy, therefore, reflects heterogeneity, historical imprint, and stabilization patterns in concept usage. A monotonic decrease in *R* signals relaxation toward the equilibrium reference, whereas increases or fluctuations indicate externally driven, reorganizing dynamics.

Thermodynamic-like quantities derived from MaxEnt formalism—generalized free energy, susceptibility, and heat capacity—provide interpretable summaries of these nonequilibrium states without attributing physical meaning to them. In particular, the identity

$$\Phi -\Phi_{\mathrm{IFP}}=TR$$

links the residual entropy to a free-energy-like measure of the informational structure, consistent with relations appearing in nonequilibrium thermodynamics [4].

An analysis of state diagrams and temporal trajectories reveals two characteristic regimes:

- Driven regime. Concepts undergo substantial variations in (β,λ), corresponding to changes in both the localization structure and the accessible frequency range. These parameter shifts generate non-adiabatic entropy production, captured by the Hatano–Sasa and Esposito–Van den Broeck decomposition. Such episodic, externally induced changes resemble jump-like updates in stochastic thermodynamics [18]. Concepts in this regime exhibit large residual entropy, *R*, and often correspond to emerging, debated, or rapidly evolving topics [22].
- Buffered (non-driven) regime. Concepts fluctuate around their equilibrium reference with monotonically decreasing residual entropy. Dissipation is dominated by the housekeeping component, and the system approaches or remains near its fixed-point distribution. Well-established, stable concepts populate this regime.

Empirically, most concepts transition from the driven to the buffered regime near an inverse-temperature value, β≈1.5, where heat capacity and susceptibility reach pronounced peaks. Around this point, the intensive entropy–energy ratio TS/E attains a value close to 1.5, consistent across years and across concepts. Concepts that approach this plateau typically enter a slow, buffered evolution, indicating that their frequency distributions have stabilized. Notably, while the intensive ratio remains approximately constant in the buffered regime, the residual free-energy gap $\Phi -\Phi_{\mathrm{IFP}}$ continues to decrease over time.

To quantify how concepts evolve across these regimes, we apply a discrete-time Hatano–Sasa/Esposito–Van den Broeck decomposition, separating entropy production into housekeeping and driving components. The resulting efficiency measures (Equations (20) and (21)) characterize how effectively a concept maintains or reorganizes its informational structure relative to total entropy production.

Most concepts operate with high housekeeping efficiency, indicating weakly driven or stabilized dynamics. Only a minority—typically those undergoing rapid semantic or topical change—exhibit substantial non-adiabatic entropy production and corresponding reductions in housekeeping efficiency. These patterns suggest that emerging or transformative concepts reside temporarily in far-from-equilibrium states before stabilizing into the buffered regime.

The framework developed here provides a unified, data-driven thermodynamic perspective on concept evolution. Pairing empirical nonequilibrium distributions with MaxEnt equilibrium references enables us to distinguish systematic structure from noise, identify stabilization and reorganization patterns, and quantify dissipation and efficiency in conceptual dynamics. Although the analogy is formal and carries no physical interpretation, the resulting thermodynamic quantities offer a coherent language for describing how scientific concepts emerge, evolve, and stabilize within the finite informational capacity of a research field.

Overall, our findings suggest that conceptual change follows measurable thermodynamic-like trajectories: from exploratory, driven evolution to buffered stability marked by entropy–energy plateaus and reduced dissipation. This perspective may support science-of-science research by identifying concepts with high transformative potential, characterizing semantic stabilization, and illuminating the collective dynamics of knowledge production.

## Data Availability

All materials related to the research are available and can be accessed through Zenodo: 10.5281/zenodo.17611701.

## Nomenclature

tf	Term frequency: number of times $k\in Z^{+}$ a concept appears in a document.
$N_{c}\left(t\right)$	Number of documents containing concept *c* up to time *t*.
$N_{c}\left(k,t\right)$	Number of documents containing *c* exactly *k* times up to time *t*.
p(k,t)	Observed probability that concept *c* is cited *k* times up to *t*.
$p_{c}\left(\left\{k\right\},t\right)\equiv p_{t}$	Mesostate probability mass function at time *t*.
$\pi^{\mathrm{eq}}\left(k,t;\beta ,\lambda \right)$	Reference probability mass function at time *t* (thermal bath).
$\pi \left(k,t\right)\equiv \pi_{t}$	Macrostate PMF or instantaneous fixed point (IFP) of the mesostate $p_{c}$.
β	Lagrange multiplier; inverse temperature ($\beta =1/(k_{B}T)$).
λ	Lagrange multiplier related to the chemical potential.
μ	Chemical potential (μ=−λ/β).
$k_{B}$	Boltzmann constant (set $k_{B}=1$ in numerical computations).
*Z*	Grand partition function that normalizes the macrostate PMF.
U=〈lnk〉	Internal (information) energy of a concept per document.
N=〈k〉	Average number of logical particles (concept mentions).
E=U−μN	The generalized energy function.
*S*	Mesostate entropy per document derived from p(k,t).
$S_{\mathrm{macro}}\equiv {§}_{\mathrm{IFP}}$	Macrostate (thermodynamic) entropy per document.
$Q^{\mathrm{eff}}=S/E$	Entropy efficiency.
*R*	Residual entropy, $R=S_{\mathrm{IFP}}-S$.
Φ	Grand canonical potential, Φ=U−TS−μN.
ΔR	Change in residual entropy over a finite interval Δt.
TR	Residual free energy ($\Phi -\Phi_{\mathrm{IFP}})$.
$C_{\mu }$	Heat capacity at constant chemical potential, $C_{\mu }={\left(\partial U/\partial T\right)}_{\mu }$.
$C_{N}$	Heat capacity at constant document support, $C_{N}={\left(\partial U/\partial T\right)}_{N}$.
$\chi_{T}$	Particle-number susceptibility, $\chi_{T}={\left(\partial N/\partial \mu \right)}_{T}$.
$\alpha_{\mu }$	Thermal expansion coefficient, $\alpha_{\mu }=\left(1/N\right){\left(\partial N/\partial T\right)}_{\mu }$.
$\alpha_{E}$	Energy–chemical potential coupling coefficient, $\alpha_{E}={\left(\partial \langle E\rangle /\partial \mu \right)}_{T}$.
$C_{\mu }^{E}$	Heat capacity at constant chemical potential for effective energy *E*.
$\eta_{\mathrm{hk}}$	Housekeeping efficiency, $\eta_{\mathrm{hk}}=\Delta S_{a}/\Delta S_{\mathrm{tot}}$.
$\eta_{\mathrm{ex}}$	Driving (adaptive) efficiency, $\eta_{\mathrm{ex}}=\Delta S_{na}/\Delta S_{\mathrm{tot}}=1-\eta_{\mathrm{hk}}$.
$\rho_{R}$	Residual-information retention ratio, $\rho_{R}=1-{\left[-\Delta R\right]}^{+}/\Delta S_{\mathrm{tot}}$.

## Appendix A. Derivation of the TR Identity for the MaxEnt Reference State

We consider MaxEnt distributions of the exponential-family form at time *t*:

(A1) $$\pi_{t}\left(k\right)=\frac{1}{Z_{t}}\exp\;\left[-\beta_{t}E_{t}\left(k\right)\right]=\frac{1}{Z_{t}}\;k^{-\beta_{t}}e^{-\lambda_{t}k},\;\lambda_{t}=\beta_{t}\mu_{t},$$

where the exponent involves the constraint function

E(k)=lnk−μk.

Let $p_{t}\left(k\right)$ denote the empirical (nonequilibrium) distribution at time *t*. We define the associated informational potential,

(A2) $$\Phi \left[p;T,\mu \right]={\langle E\rangle }_{p}-T\;S\left[p\right]=\sum_{k}p_{k}\left(\ln k-\mu k\right)-T(-\sum_{k}p_{k}\ln p_{k}),$$

and its MaxEnt (IFP) counterpart,

(A3) $$\Phi_{\mathrm{IFP}}\left(T,\mu \right)=\Phi \left[\pi ;T,\mu \right]=-\;T\ln Z.$$

For any *p* and its corresponding IFP π at the same (T,μ),

(A4) $$\Phi \left[p;T,\mu \right]-\Phi_{\mathrm{IFP}}\left(T,\mu \right)=T\;D_{\mathrm{KL}}\left(p∥\pi \right)=T\;R\left(p∥\pi \right)\geq 0,$$

with equality only when p=π.

**Proof.** The Kullback–Leibler divergence is

$$R\left(p∥\pi \right)=\sum_{k}p_{k}\ln\frac{p_{k}}{\pi_{k}}=\sum_{k}p_{k}\ln p_{k}-\sum_{k}p_{k}\ln\pi_{k}.$$

Since $\ln\pi_{k}=-\beta E\left(k\right)-\ln Z$, we obtain

$$R\left(p∥\pi \right)=\sum_{k}p_{k}\ln p_{k}+\beta \sum_{k}p_{k}E\left(k\right)+\ln Z.$$

Multiplying by *T* and using Tβ=1 yields

$$TR=T\sum_{k}p_{k}\ln p_{k}+\sum_{k}p_{k}E\left(k\right)+T\ln Z=\Phi \left[p;T,\mu \right]-\Phi_{\mathrm{IFP}}\left(T,\mu \right).$$

  □

The identity (A4) requires only that (T,μ) be fixed; it does not assume fixed moments such as 〈k〉 or 〈lnk〉. Differences in these moments are fully encoded in the KL divergence.

On a discrete time grid, $\left\{t_{n}\right\}$,

(A5) $$\Phi \left(p_{t_{n}}\right)-\Phi_{\mathrm{IFP}}\left(t_{n}\right)=T_{t_{n}}\;R\left(p_{t_{n}}∥\pi_{t_{n}}\right)\geq 0.$$

Thus, the decomposition

(A6) $$\Phi \left[p;T,\mu \right]=\Phi_{\mathrm{IFP}}\left(T,\mu \right)+T\;R\left(p∥\pi \right)$$

makes explicit the contribution of the residual information *R* as the nonequilibrium excess over the MaxEnt reference.

## Appendix B. Irreversible Work Identity for Informational Grand Potentials

The purpose of this appendix is to show that, for exponential family (MaxEnt) distributions with constrained observables 〈lnk〉 and 〈k〉, the algebraic structure of the Legendre transform leads to an identity formally equivalent to the nonequilibrium free–energy relations derived by Esposito and Van den Broeck [4].

We define the informational grand potential

$$\Phi_{t}\equiv U_{t}-T_{t}S_{t}-\mu_{t}N_{t},$$

where $U_{t}={\langle \ln k\rangle }_{p_{t}}$, $N_{t}={\langle k\rangle }_{p_{t}}$, and $S_{t}$ is the Shannon entropy. Differentiating gives the exact identity

(A7) $$d\Phi_{t}=dU_{t}-T_{t}\;dS_{t}-S_{t}\;dT_{t}-\mu_{t}\;dN_{t}-N_{t}\;d\mu_{t}.$$

In classical nonequilibrium thermodynamics [47,48], entropy changes are decomposed as

$$dS_{t}=dS_{e,t}+dS_{i,t},\;dS_{i,t}\geq 0,$$

where $dS_{e,t}$ is the reversible contribution induced via the protocol, and $dS_{i,t}$ is entropy production.

To ensure consistency with the Legendre structure of $\Phi_{t}$, we define the reversible part using the identity

$$T_{t}\;dS_{e,t}\equiv dU_{t}-\mu_{t}dN_{t},$$

which reduces to the usual reversible relation when $p_{t}=\pi_{t}$. Thus,

(A8) $$T_{t}\;dS_{i,t}\equiv dU_{t}-\mu_{t}dN_{t}-T_{t}\;dS_{t}.$$

Insert Equation (A8) into the differential of $\Phi_{t}$ (Equation (A7)), and simplify. Introducing the formal forcing term

$$\delta W_{\mathrm{form}}\equiv dU_{t}-\mu_{t}dN_{t},$$

one obtains the exact identity

(A9) $$T_{t}\;dS_{i,t}=\delta W_{\mathrm{form}}-d\Phi_{t}-S_{t}\;dT_{t}-N_{t}\;d\mu_{t}.$$

Equation (A9) is a formal rewriting of the Hatano–Sasa/Esposito decomposition applied to the informational potential, rather than to a physical free energy.

Differentiating the identity $\Phi_{t}=\Phi_{\mathrm{IFP},t}+T_{t}R_{t}$ yields

(A10) $$d\Phi_{t}=d\Phi_{\mathrm{IFP},t}+T_{t}\;dR_{t}+R_{t}\;dT_{t}.$$

Substituting Equation (A10) into Equation (A9) gives

$$T_{t}\;dS_{i,t}=\delta W_{\mathrm{form}}-d\Phi_{\mathrm{IFP},t}-T_{t}\;dR_{t}-R_{t}\;dT_{t}-S_{t}\;dT_{t}-N_{t}\;d\mu_{t}.$$

Integrating from $t_{i}\;$ to $t_{f}$ gives the total irreversibility:

(A11) $$W_{\mathrm{irr}}\equiv \int_{t_{i}}^{t_{f}}\delta W_{\mathrm{form}}-W_{\mathrm{rev}}=\int_{t_{i}}^{t_{f}}T_{t}\;dS_{i,t}+\int_{t_{i}}^{t_{f}}T_{t}\;dR_{t},$$

where $W_{\mathrm{rev}}$ denotes the reversible contribution obtained when the protocol is restricted to the IFP manifold ($R_{t}=0$, $S_{i,t}=0$).

For protocols that keep (T,μ) fixed, Equation (A11) reduces to

(A12) $$W_{\mathrm{irr}}=T\;\Delta S_{i}+T\;\Delta R.$$

Equation (A12) is the *informational* analog of the iso–(T,μ) irreversible work identity in nonequilibrium thermodynamics [4]. Here, $T\;\Delta S_{i}$ measures irreversible dispersion generated via the protocol, and TΔR measures the change in the nonequilibrium structure relative to the MaxEnt baseline.

## Appendix C. Entropy Production Under Instantaneous Fixed-Point Reference

In this appendix, we demonstrate that, when the reference distribution is chosen as the instantaneous fixed point of the system, the time derivative of the residual entropy equals the negative of the entropy production rate:

(A13) $$\dot{R}\left(t\right)=-{\dot{S}}_{i}\left(t\right).$$

Let $p_{i}\left(t\right)$ denote the empirical (mesoscopic) probability of observing the microstate *i* at time *t*, corresponding in the main text to the document–level frequency distribution p(k,t) defined in Equation (1). The instantaneous fixed point or macrostate of the system is the distribution that maximizes the Shannon entropy $S_{\mathrm{macro}}\left(t\right)=-\sum_{i}\pi_{i}\left(t\right)\ln\pi_{i}\left(t\right)$ under the current empirical constraints ${\langle f_{j}\rangle }_{p(t)}={\langle f_{j}\rangle }_{\pi (t)}.$ This constrained maximization yields an exponential–family form (cf. Equations (2) and (3) in the main text):

(A14) $$\pi_{i}\left(t\right)=\frac{1}{Z(t)}\exp\;\left[-\sum_{j}\theta_{j}\left(t\right)f_{j}\left(i\right)\right],\;Z\left(t\right)=\sum_{i}\exp\;\left[-\sum_{j}\theta_{j}\left(t\right)f_{j}\left(i\right)\right],$$

where the Lagrange multipliers $\theta_{j}\left(t\right)$ (identified in the main text as β(t) and λ(t)) are determined at each instant by the empirical moments ${\langle f_{j}\rangle }_{p(t)}$.

The residual entropy—the informational distance between the empirical distribution and its instantaneous MaxEnt reference—is the Kullback–Leibler divergence (cf. Equation (6) in the main text):

(A15) $$R\left(t\right)=D_{\mathrm{KL}}\;\left(p(t)\;∥\;\pi (t)\right)=\sum_{i}p_{i}\left(t\right)\;\ln\frac{p_{i}\left(t\right)}{\pi_{i}\left(t\right)}\geq 0.$$

The equality R=0 occurs only when the mesoscopic state coincides with its IFP, p(t)=π(t), that is, when the system is locally in equilibrium with respect to the chosen constraints.

Taking the time derivative of Equation (A15) gives

(A16) $$\begin{array}{cc} \dot{R}\left(t\right) & =\;\sum_{i}{\dot{p}}_{i}\ln\frac{p_{i}}{\pi_{i}}+\sum_{i}p_{i}\;\frac{d}{dt}\;\left[\ln\frac{p_{i}}{\pi_{i}}\right]\\ \; & =\;\sum_{i}{\dot{p}}_{i}\ln\frac{p_{i}}{\pi_{i}}+\sum_{i}p_{i}\;\left(\frac{{\dot{p}}_{i}}{p_{i}}-\frac{{\dot{\pi }}_{i}}{\pi_{i}}\right). \end{array}$$

The first sum in the second term vanishes because the probabilities are normalized, $\sum_{i}{\dot{p}}_{i}=0$. Hence,

(A17) $$\dot{R}\left(t\right)=\sum_{i}{\dot{p}}_{i}\ln\frac{p_{i}}{\pi_{i}}-\sum_{i}p_{i}\;\frac{{\dot{\pi }}_{i}}{\pi_{i}}.$$

From Equation (A14), we have $\ln\pi_{i}=-\ln Z-\sum_{j}\theta_{j}f_{j}\left(i\right)$, so that

(A18) $$\frac{{\dot{\pi }}_{i}}{\pi_{i}}=-\partial_{t}\ln Z-\sum_{j}{\dot{\theta }}_{j}\;f_{j}\left(i\right).$$

Taking the expectation value with respect to the empirical distribution $p_{i}$:

(A19) $$\begin{array}{cc} \sum_{i}p_{i}\frac{{\dot{\pi }}_{i}}{\pi_{i}} & =\;-\partial_{t}\ln Z-\sum_{j}{\dot{\theta }}_{j}\;{\langle f_{j}\rangle }_{p}. \end{array}$$

For an exponential-family distribution, the partition function satisfies $\partial_{\theta_{j}}\ln Z=-{\langle f_{j}\rangle }_{\pi }$, so that $\partial_{t}\ln Z=\sum_{j}\partial_{\theta_{j}}\ln Z\;{\dot{\theta }}_{j}=-\sum_{j}{\dot{\theta }}_{j}\;{\langle f_{j}\rangle }_{\pi }$. Substituting this into Equation (A19) gives

$$\sum_{i}p_{i}\;\frac{{\dot{\pi }}_{i}}{\pi_{i}}=-\sum_{j}{\dot{\theta }}_{j}\;\left({\langle f_{j}\rangle }_{p}-{\langle f_{j}\rangle }_{\pi }\right).$$

By definition of the instantaneous fixed point, these expectation values coincide (${\langle f_{j}\rangle }_{p}={\langle f_{j}\rangle }_{\Pi }$), and therefore,

(A20) $$\sum_{i}p_{i}\;\frac{{\dot{\pi }}_{i}}{\pi_{i}}=0.$$

With Equation (A20), the residual-entropy rate (A17) reduces to

(A21) $$\dot{R}\left(t\right)=\sum_{i}{\dot{p}}_{i}\left(t\right)\;\ln\frac{p_{i}\left(t\right)}{\pi_{i}\left(t\right)}.$$

For a continuous-time Markov process with transition rates $W_{ij}$, the entropy production rate is defined as [18]

(A22) $${\dot{S}}_{i}\left(t\right)=\frac{1}{2}\sum_{i,j}J_{ij}\left(t\right)\;\ln\frac{p_{i}\left(t\right)W_{ij}}{p_{j}\left(t\right)W_{ji}},\;J_{ij}=p_{i}W_{ij}-p_{j}W_{ji}.$$

If the transition rates satisfy detailed balance with respect to the instantaneous reference distribution $\pi_{i}\left(t\right)$,

$$\pi_{i}\left(t\right)\;W_{ij}\left(t\right)=\pi_{j}\left(t\right)\;W_{ji}\left(t\right),$$

then

$$\begin{array}{cc} {\dot{S}}_{i}\left(t\right) & =\;\frac{1}{2}\sum_{i,j}\left(p_{i}W_{ij}-p_{j}W_{ji}\right)\ln\;\left(\frac{p_{i}W_{ij}}{p_{j}W_{ji}}\right)\\ \; & =\;\frac{1}{2}\sum_{i,j}\left(p_{i}W_{ij}-p_{j}W_{ji}\right)\ln\;\left(\frac{p_{i}/\pi_{i}}{p_{j}/\pi_{j}}\right). \end{array}$$

Define $f_{i}=p_{i}/\pi_{i}$. Using the antisymmetry of $J_{ij}=p_{i}W_{ij}-p_{j}W_{ji}$ and the symmetry of the sum, we obtain the following:

(A23) $${\dot{S}}_{i}\left(t\right)=\sum_{i,j}p_{i}W_{ij}\ln\;\left(\frac{f_{i}}{f_{j}}\right).$$

Now, consider the expression $-\sum_{i}{\dot{p}}_{i}\ln f_{i}$. Using the master equation ${\dot{p}}_{i}=\sum_{j}\left(p_{j}W_{ji}-p_{i}W_{ij}\right)$:

$$\begin{array}{cc} -\sum_{i}{\dot{p}}_{i}\ln f_{i} & =\;-\sum_{i,j}\ln f_{i}\left(p_{j}W_{ji}-p_{i}W_{ij}\right)\\ \; & =\;\sum_{i,j}p_{i}W_{ij}\ln f_{i}-\sum_{i,j}p_{j}W_{ji}\ln f_{i}. \end{array}$$

We relabel indices i↔j in the second sum:

$$-\sum_{i}{\dot{p}}_{i}\ln f_{i}=\sum_{i,j}p_{i}W_{ij}\left(\ln f_{i}-\ln f_{j}\right)=\sum_{i,j}p_{i}W_{ij}\ln\;\left(\frac{f_{i}}{f_{j}}\right).$$

We can see that this matches Equation (A23) for ${\dot{S}}_{i}\left(t\right)$. Therefore, the right-hand side of Equation (A22) can be rewritten as

$${\dot{S}}_{i}\left(t\right)=-\sum_{i}{\dot{p}}_{i}\left(t\right)\;\ln\frac{p_{i}\left(t\right)}{\pi_{i}\left(t\right)}.$$

Comparing with Equation (A21) immediately yields the desired identity

(A24) $$\dot{R}\left(t\right)=-\;{\dot{S}}_{i}\left(t\right).$$

Equation (A24) shows that, when the reference state is the instantaneous MaxEnt fixed point π(t) (determined self-consistently from the data at each time), the residual entropy R(t) decreases exactly at the rate at which the entropy is produced. In fact, the reference state need not be the IFP of the empirical mesostate; Equation (A21) holds when $\dot{\pi }=0$ and its temperature and chemical potential remained constant (${\dot{\theta }}_{j}=0$) for the reference state. To see this, we need to compare Equations (A21) and (A32).

In both cases, the system relaxes monotonically toward π, and the relative entropy R(t) plays the role of a Lyapunov function:

$$\dot{R}\left(t\right)\leq 0,\;{\dot{S}}_{i}\left(t\right)\geq 0.$$

The identification of *R* as a measure of “informational distance from equilibrium” justifies its interpretation as an internal measure of the system’s nonequilibrium organization, and it explains why, under IFP dynamics, entropy production precisely quantifies the rate of information dissipation.

**Mid-point approximation for an entropy-production estimate.** In the main text, we distinguish two informational quantities: (i) the residual entropy $R\left(t\right)=D_{\mathrm{KL}}\left(p_{t}∥\pi_{t}\right)$, which measures the instantaneous separation between the empirical mesostate $p_{t}$ and its MaxEnt reference (IFP) $\pi_{t}$, and (ii) the entropy production $\Delta S_{i}$, which measures the total irreversibility accumulated along the transition between two time points. These quantities coincide only in the special case of relaxation toward a *fixed* reference distribution; in general, *R* is a state function (defined at each *t*), while $\Delta S_{i}$ is path-dependent because the reference $\pi_{t}$ changes over time.

To formalize this distinction without invoking physical heat flows, we use the standard Hatano–Sasa/Esposito–Van den Broeck informational balance for a time-dependent reference distribution $\pi_{t}$:

(A25) $$\Delta S_{i}=\Delta S+\Delta S_{\mathrm{ex}},\;\Delta S\equiv S\left[p_{t_{f}}\right]-S\left[p_{t_{i}}\right],$$

where the *excess* contribution is defined as

(A26) $$\Delta S_{\mathrm{ex}}\;\equiv \;-\int_{t_{i}}^{t_{f}}\;dt\;\sum_{k}p\left(k,t\right)\;\partial_{t}\ln\pi \left(k,t\right).$$

Equation (A25) is the informational analogue of the entropy production decomposition used in stochastic thermodynamics: $\Delta S_{i}$ captures irreversibility, while $\Delta S_{\mathrm{ex}}$ accounts for the fact that the reference (IFP) itself moves in time under the external protocol.

Our data are available only at discrete times $t_{i}$ and $t_{f}$. We therefore approximate the integral in Equation (A26) by a midpoint (Stratonovich) rule [49], i.e.,

(A27) $$\Delta S_{\mathrm{ex}}\;\approx \;-\sum_{k}p_{*}\left(k\right)\;\left[\ln\pi_{f}\left(k\right)-\ln\pi_{i}\left(k\right)\right],\;p_{*}\left(k\right)\equiv \frac{1}{2}\left[p_{i}\left(k\right)+p_{f}\left(k\right)\right],$$

where $p_{i}\equiv p\left(\cdot ,t_{i}\right)$ and $p_{f}\equiv p\left(\cdot ,t_{f}\right)$, and likewise $\pi_{i}\equiv \pi \left(\cdot ,t_{i}\right)$, $\pi_{f}\equiv \pi \left(\cdot ,t_{f}\right)$.

In our MaxEnt construction the IFP has the generalized Boltzmann form $\pi \left(k,t\right)\propto k^{-\beta (t)}e^{-\lambda (t)k}$. To implement the midpoint rule consistently at the level of the protocol parameters, we also introduce midpoint intensives

(A28) $$\beta_{*}=\frac{1}{2}\left[\beta \left(t_{i}\right)+\beta \left(t_{f}\right)\right],\;\mu_{*}=-\;\frac{\lambda \left(t_{i}\right)+\lambda \left(t_{f}\right)}{\beta \left(t_{i}\right)+\beta \left(t_{f}\right)},$$

which correspond to the midpoint of (β,μ) under the identity μ(t)=−λ(t)/β(t). (Equivalently, one may work with (β,λ) directly; we use (β,μ) only to match the grand-canonical notation adopted in the main text.)

Finally, combining Equations (A25) and (A27), we obtain the discrete-time estimate used in the Results:

(A29) $$\Delta S_{i}\;\approx \;\left[S\left[p_{f}\right]-S\left[p_{i}\right]\right]-\sum_{k}p_{*}\left(k\right)\;\left[\ln\pi_{f}\left(k\right)-\ln\pi_{i}\left(k\right)\right].$$

This estimator makes explicit why $\Delta S_{i}$ is not, in general, equal to ΔR or to the endpoint KL divergences: it depends on the *change of the reference* along the interval, approximated here by a midpoint rule.

## Appendix D. Dynamic Efficiency and Work Decomposition in Concept Evolution

We present a derivation of the discrete-time decomposition of entropy production into non-adiabatic (excess) and adiabatic (housekeeping) parts, as given by Equations (18) and (17) of the manuscript. This derivation follows the frameworks of Hatano and Sasa [30] and Esposito–Van der Broeck [15].

In stochastic thermodynamics, the total entropy production rate ${\dot{S}}_{\mathrm{tot}}\left(t\right)$ can be split into two non-negative contributions:

(A30) $${\dot{S}}_{\mathrm{tot}}={\dot{S}}_{\mathrm{na}}+{\dot{S}}_{a}.$$

Here, ${\dot{S}}_{\mathrm{na}}$ is the non-adiabatic (excess) entropy production rate due to the change of control parameters, and ${\dot{S}}_{a}$ is the adiabatic (housekeeping) entropy production rate required to maintain the non-equilibrium steady state, even when the control parameters are fixed.

Following [15], the non-adiabatic part can be expressed as

(A31) $${\dot{S}}_{\mathrm{na}}=-\frac{d}{dt}D\left(p_{t}∥\pi_{t}\right)+{\dot{S}}_{\mathrm{ex}},$$

where the excess entropy production rate ${\dot{S}}_{\mathrm{ex}}$ is given by

(A32) $${\dot{S}}_{\mathrm{ex}}=-\sum_{k}p_{t}\left(k\right)\;\partial_{t}\ln\pi_{t}\left(k\right).$$

Integrating Equation (A31) from an initial time, $t_{i}$, to a final time, $t_{f}$, yields

(A33) $$\Delta S_{\mathrm{na}}=-[D\left(p_{t_{f}}∥\pi_{t_{f}}\right)-D\left(p_{t_{i}}∥\pi_{t_{i}}\right)]+\int_{t_{i}}^{t_{f}}{\dot{S}}_{\mathrm{ex}}\;dt=-\Delta R+\Delta S_{\mathrm{ex}},$$

with $\Delta R=R_{t_{f}}-R_{t_{i}}$ (see Equation (6) from the main text) and $\Delta S_{\mathrm{ex}}=\int_{t_{i}}^{t_{f}}{\dot{S}}_{\mathrm{ex}}\;dt$.

To obtain a form suitable for the analysis of discrete-time data, we approximate the integral of ${\dot{S}}_{\mathrm{ex}}$ using a sum over discrete time steps. Let the time interval be divided into steps of size Δt=1 (without a loss in generality). For a step from *t* to t+1, we approximate the excess entropy production using the functional introduced by Hatano and Sasa [30], which, in our notations, reads as $\phi_{t}\left(k\right)=-\ln\pi_{t}\left(k\right)$. For a discrete-time trajectory $p_{t}\;\to \;p_{t+1}$, we define the excess functional

(A34) $${\hat{Y}}_{t\to t+1}=\sum_{k}p_{t}\left(k\right)\;\left[\phi_{t+1}\left(k\right)-\phi_{t}\left(k\right)\right]=\sum_{k}p_{t}\left(k\right)\;\ln\frac{\pi_{t}\left(k\right)}{\pi_{t+1}\left(k\right)},$$

which satisfies $\langle e^{-\hat{Y}}\rangle =1$ and, therefore, $\langle \hat{Y}\rangle \geq 0$.

To see the connection, note that, in the continuous limit,

$$\ln\frac{\pi_{t}\left(k\right)}{\pi_{t+1}\left(k\right)}\approx -\partial_{t}\ln\pi_{t}\left(k\right)\;\Delta t,$$

so that

$${\hat{Y}}_{t\to t+1}\approx \Delta t\;{\dot{S}}_{\mathrm{ex}}\left(t\right).$$

Summing over all steps from $t_{i}$ to $t_{f}-1$ gives

(A35) $$\sum_{t=t_{i}}^{t_{f}-1}{\hat{Y}}_{t\to t+1}\approx \int_{t_{i}}^{t_{f}}{\dot{S}}_{\mathrm{ex}}\;dt=\Delta S_{\mathrm{ex}}.$$

Substituting this discrete approximation into Equation (A33) yields

(A36) $$\Delta S_{\mathrm{na}}\approx -\Delta R+\sum_{t=t_{i}}^{t_{f}-1}{\hat{Y}}_{t\to t+1}>0.$$

The adiabatic (housekeeping) entropy production is then the remainder:

(A37) $$\Delta S_{a}=\Delta S_{\mathrm{tot}}-\Delta S_{\mathrm{na}}\geq 0.$$
